# Draft genome sequence and overview of the purple non sulfur bacterium *Rhodopseudomonas palustris* 42OL

**DOI:** 10.1186/s40793-016-0145-y

**Published:** 2016-03-09

**Authors:** Alessandra Adessi, Giulia Spini, Luana Presta, Alessio Mengoni, Carlo Viti, Luciana Giovannetti, Renato Fani, Roberto De Philippis

**Affiliations:** Department of Agrifood Production and Environmental Sciences, University of Florence, via Maragliano 77, 50144 Firenze, Italy; Institute of Chemistry of Organometallic Compounds, National Research Council, Via Madonna del Piano 10, 50019 Sesto Fiorentino, Italy; Department of Biology, University of Florence, Via Madonna del Piano 6, 50019 Sesto Fiorentino, Italy

**Keywords:** *Rhodopseudomonas palustris* 42OL, Purple non-sulfur bacteria, Hydrogen production, Wastewater treatment, PHB accumulation

## Abstract

*Rhodopseudomonas palustris* strain 42OL was isolated in 1973 from a sugar refinery waste treatment pond. The strain has been prevalently used for hydrogen production processes using a wide variety of waste-derived substrates, and cultured both indoors and outdoors, either freely suspended or immobilized. *R. palustris* 42OL was suitable for many other applications and capable of growing in very different culturing conditions, revealing a wide metabolic versatility. The analysis of the genome sequence allowed to identify the metabolic pathways for hydrogen and poly-β-hydroxy-butyrate production, and confirmed the ability of using a wide range of organic acids as substrates.

## Introduction

*Rhodopseudomonas palustris* is a PNSB belonging to the class *Alphaproteobacteria*. According to Imhoff et al. [1], the term PNSB is used to indicate a physiological group of anoxygenic phototrophic bacteria, affiliated to both *Alphaproteobacteria* and *Betaproteobacteria*, containing photosynthethic pigments and able to carry out anoxygenic photosynthesis.

Strains of *R. palustris* have been isolated from a variety of different environments, from eutrophic lagoons to moist soils, from freshwater ponds to marine coastal sediments [2–4]. The very wide spread of *R. palustris* throughout a variety of habitats is due to its extreme metabolic versatility, with all modes of metabolism represented (autotrophic, heterotrophic, organotrophic, litotrophic, chemotrophic and phototrophic); moreover, the organism is a facultative anaerobe [5].

All PNSBs are characterized by the ability of carrying out anoxygenic photosynthesis; in the presence of oxygen, photosynthesis is inhibited and a number of PNSBs are able to carry out respiration [4]. Under anaerobic conditions, and subject to light irradiation, PNSBs are able to fix nitrogen via nitrogenase; hydrogen is produced as a by-product of nitrogen fixation. Among PNSBs, *R. palustris* is considered a model organism for studying biological hydrogen production, due to its capacity of efficiently producing hydrogen during organic wastes degradation [6].

*R. palustris* 42OL has been used previously for hydrogen production processes under various conditions [7–22], i.e., with different substrates, and cultured indoors and outdoors, using freely suspended or immobilized cells. However, its first application was in mixed culture in wastewater treatment [23]. Its biomass was evaluated for SCP accumulation and amino acid composition [24]. The accumulation of PHB and its connection to hydrogen production were investigated [8, 11, 25]. More recently, the biomass of *R. palustris* 42OL was also used as a biosorbent for metal removal from wastewaters [26, 27]. Furthermore, the NMR and X-ray structures of its 7Fe-8S ferredoxin and cytochrome *c*_2_ were studied [28–30]. The latest application of the strain was for antibiotic delivery though liposomes formed with its lipids [31].

The long history and the versatility of this particular strain render it a very good candidate for further investigating the basis of its ability to acclimate to very different culturing conditions.

## Organism information

### Classification and features

*R. palustris* 42OL was isolated in 1973 from a catch-basin collecting the effluents of a sugar refinery waste treatment pond, in Castiglion Fiorentino (AR), Italy. The enrichment was carried out aimed at selecting waste degrading phototrophs. The isolated microorganism has been stored since 1973 at CSMA Collection (WDCM number 147) under the collection name CSMA73/42, growing anaerobically on solid RPN medium [32] with malate 2 g L^-1^ as the carbon source and supplemented with 0.4 g L^-1^ of yeast extract. The general features of the isolate are reported in Table 1.

**Table 1:** Classification and general features of *Rhodopseudomonas palustris* 42OL, according to MIGS standards [45]

MIGS ID	Property	Term	Evidence code^a^
	Classification	Domain *Bacteria*	TAS [46]
		Phylum *Proteobacteria*	TAS [47]
		Class *Alphaproteobacteria*	TAS [48, 49]
		Order *Rhizobiales*	TAS [49, 50]
		Family *Bradyrhizobiaceae*	TAS [49, 50]
		Genus *Rhodopseudomonas*	TAS [1, 51, 52]
		Species *Rhodopseudomonas palustris*	TAS [51, 53, 54]
		strain: 42OL *(CSMA73/42)*	
	Gram stain	Negative	NAS
	Cell shape	Rod	IDA
	Motility	Motile only during first part of cell cycle	NAS
	Sporulation	Non sporulating	
	Temperature range	mesophilic	NAS
	Optimum temperature	28–30 °C	IDA
	pH range; Optimum	6.0–8.0; 6.8	TAS [32]
	Carbon source	VFA, CO_2_	IDA
MIGS-6	Habitat	Sugar refinery waste pond	IDA
MIGS-6.3	Salinity	Not determined	
MIGS-22	Oxygen requirement	Facultatively anaerobic	IDA
MIGS-15	Biotic relationship	Free-living	NAS
MIGS-14	Pathogenicity	Non-pathogen	NAS
MIGS-4	Geographic location	Castiglion Fiorentino, AR, Italy	IDA
MIGS-5	Sample collection	1973	IDA
MIGS-4.1	Latitude	43° 19' 30.054"	IDA
MIGS-4.2	Longitude	11° 53' 18.4518"	IDA
MIGS-4.4	Altitude	248 m	IDA

^a^Evidence codes - *IDA* inferred from direct assay, *TAS* traceable author statement (i.e., a direct report exists in the literature), *NAS* non-traceable author statement (i.e., not directly observed for the living, isolated sample, but based on a generally accepted property for the species, or anecdotal evidence). These evidence codes are from the Gene Ontology project [55]

**Fig. 1:**
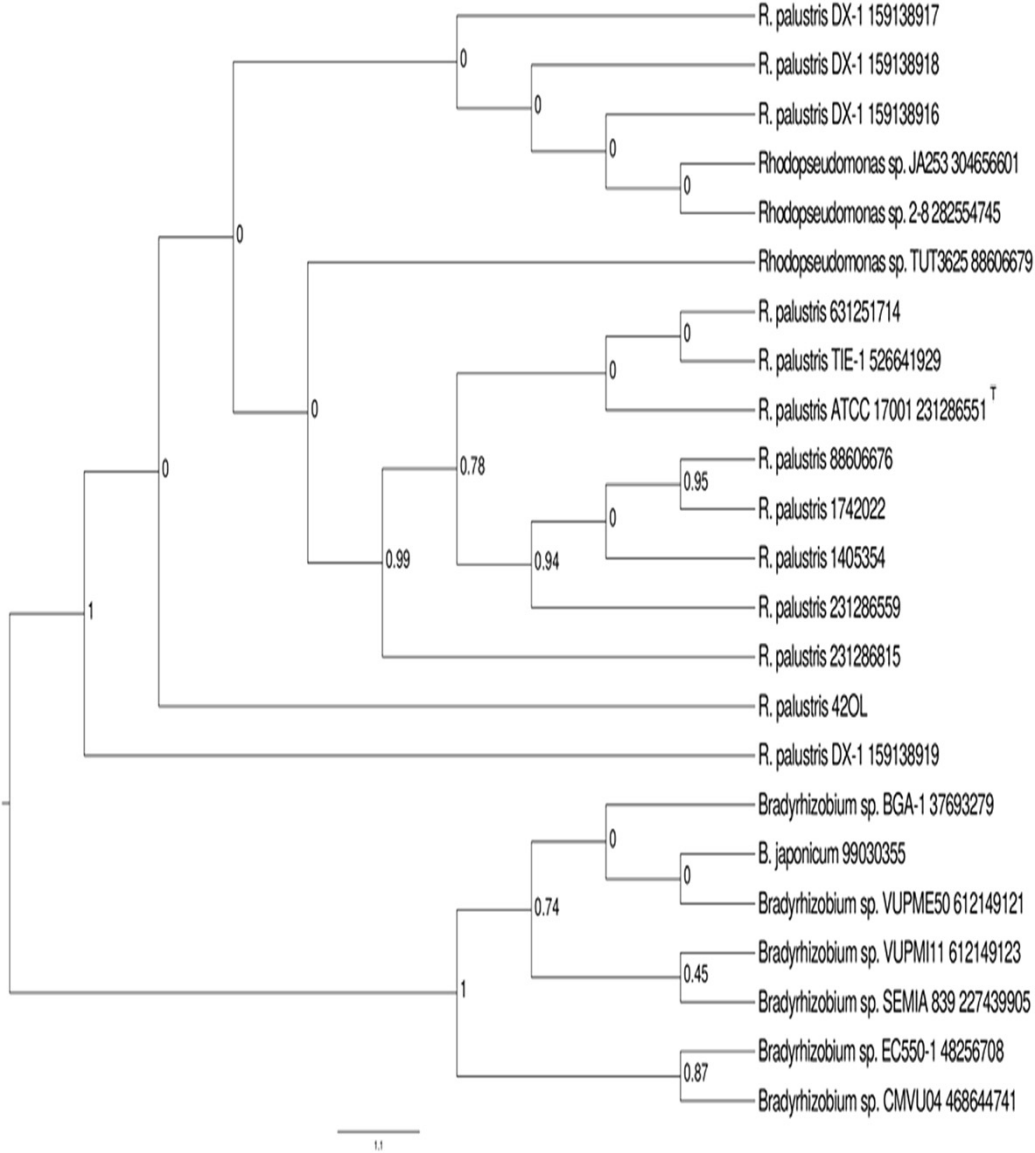
Maximum Likelihood dendrogram based on 16S rRNA gene; Bar = 1.1 indicates the nucleotides substitution rate. Numbers at the nodes indicate bootstrap values after 500 random replicates. Numbers after strain name indicate the GI code. Sequences have been selected after BLAST Explorer [56] search for most similar sequences present in GenBank database. Dendrogram has been constructed by using the Maximum Likelihood algorithm with default options present in phylogeny.fr web server [56]. Strains TIE-1 and DX-1 have completely sequenced genomes; ATCC17001 is the type strain and is indicated as^T^

The isolate 42OL was firstly assigned morphologically to *R. palustris*. Phylogenetic analysis performed subsequently (unpublished results) by comparing 16S rRNA gene sequences revealed that the isolate might be indeed affiliated to the species *R. palustris*. With the present work, a further phylogenetic analysis was conducted and, as shown in the phylogenetic tree in Fig. 1, confirms its affiliation.

**Fig. 2:**
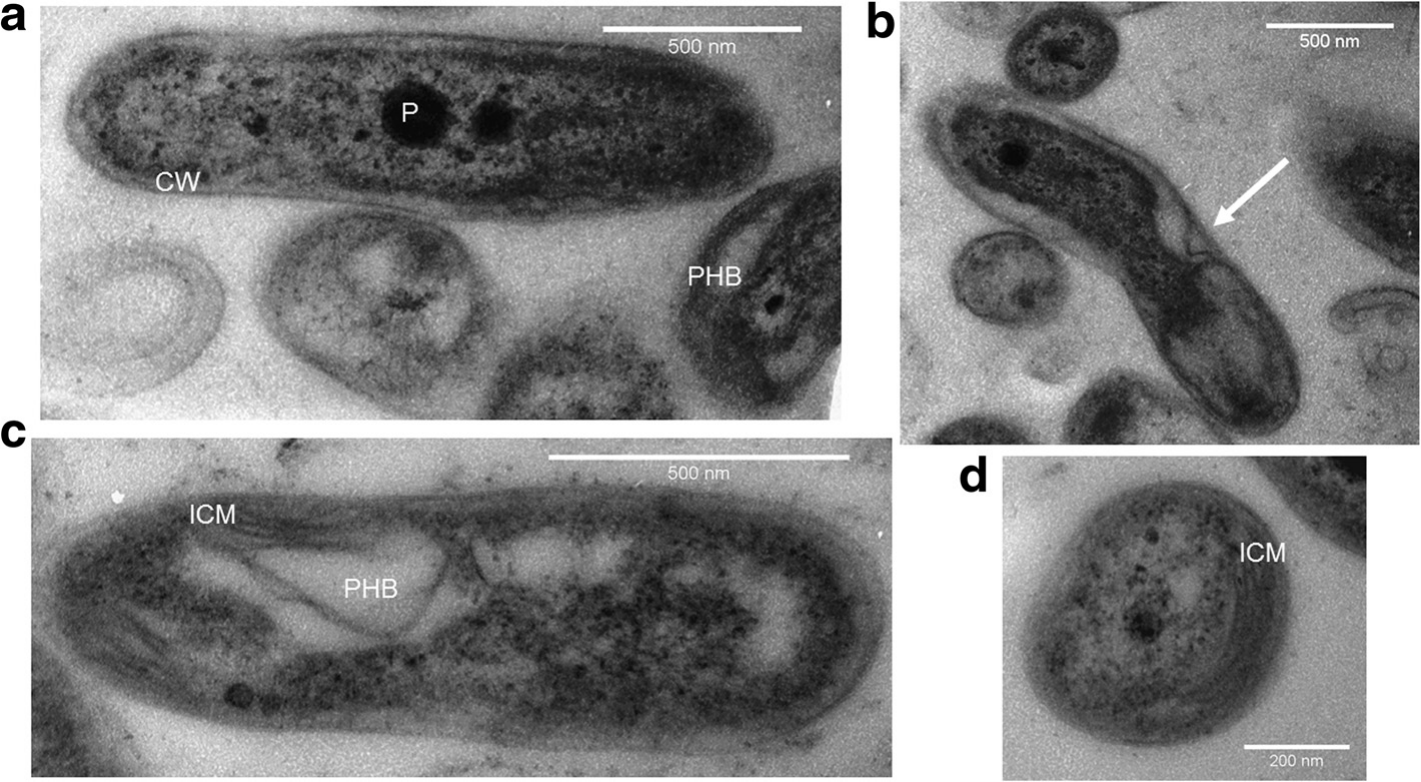
Electron micrographs of *Rhodopseudomonas palustris* 42OL grown on RPN medium; **a** whole cell, longitudinal section; **b** cell during polar budding (white arrow), longitudinal section; **c** whole cell containing PHB granules. **d** lamellar ICMs in whole cells, transversal section; PHB, poly-β-hydroxybutyrate granules; ICM, intra-cytoplasmic membranes; P, polyphosphate granules; CW, cell wall

*R. palustris* 42OL is a Gram-negative rod shaped bacterium, of 0.6–1.2 μm (see Fig. 2a). It replicates by polar budding (Fig. 2b) and new cells present one single *flagellum* that is lost in the subsequent phases of cell cycle [33, 34]. Its photosynthetic apparatus is located on lamellar ICMs, clearly visible in Fig. 2c, d, as characteristic of this species [2]. The major carotenoid molecules that are present in the photosynthetic apparatus of this strain are spirilloxanthin, rhodopin, rhodovibrin, anhydrorhodovibrin and lycopene (our unpublished results).

The first characterization of *R. palustris* 42OL was in terms of protein accumulation and amino acid composition on different carbon and nitrogen sources. Malate and ethanol were tested as carbon sources, both under nitrogen fixing (N_2_ sparged) and non-fixing (NH_4_^+^ supplied) conditions. A significantly lower amount of protein was produced in presence of ethanol, while the nitrogen source did not have any effect. However, both nitrogen and carbon sources significantly influenced the amino acid composition of the protein biomass [24].

The carbon metabolism of the strain was investigated in terms of substrates that could be used for growth and hydrogen production. Short chain fatty acids such as acetate, pyruvate, lactate, malate and succinate were found to be photodissimilated by the strain with substrate conversion efficiency of 40, 52, 61, 56, and 67 %, respectively [35]. Butyrate was found to be suitable for growth and hydrogen production but with the significantly lower substrate-to-hydrogen conversion efficiency of 9 % (unpublished data).

Vincenzini et al. [36] characterized nitrogenase activity of *R. palustris* 42OL in terms of hydrogen production and acetylene reduction with different atmosphere composition and at different pH values. The optimal conditions for hydrogen production were under Argon gas as the atmosphere, for early logarithmic stage cells, at pH 6.8. The authors also demonstrated the presence of a hydrogenase enzyme, recycling the hydrogen produced during late logarithmic and light-limited stage.

Evidences of its suitability for the treatment of wastes combined with hydrogen production were reported [35], using wastewaters deriving from a sugar refinery and a paper mill. The same strain was shown to grow and produce hydrogen on different substrates such as vegetable wastes [15], olive mill wastewaters [12, 13] and dark fermentation saline effluents [14].

PHB is synthetized as a reservoir for reducing equivalents by the strain, in this way competing with hydrogen production [25], especially when grown on acetate [25], or when subject to phosphorus starvation [11]; in this condition, *R. palustris* 42OL could accumulate up to 18 % w/w of PHB on cell dry weight. *R. palustris* 42OL accumulates PHB in large amorphous granules, as shown in Fig. 2. Glycogen is synthetized as well as carbon and energy reserve [25].

Another relevant characteristic of this strain is the possibility of cultivation outdoors, under light/dark cycles both for biomass [37, 38] and hydrogen production [10, 11, 22], with an impressive capability of the photosystem to take advantage of the high light conditions that take place during the central hours of the day [21].

## Genome sequencing information

### Genome project history

The organism was selected for genome sequencing on the basis of its metabolic versatility and biotechnological relevance, as witnessed by its long history and by the diversity of applications. Project information is available from the Genomes OnLine Database [39], under the GOLD study ID Gs0114708. The WGS sequence is deposited in GenBank (LCZM00000000).

### Growth conditions and genomic DNA preparation

*R. palustris* 42OL (CSMA73/42) was maintained anaerobically on solid RPN medium [32] with malate 2 g L^-1^ as the carbon source and supplemented with 0.4 g L^-1^ of yeast extract. For the extraction of genomic DNA a single colony of cells grown on agar plate was harvested and cultured anaerobically on the same liquid medium in 20 mL sealed glass tubes, at room temperature with a light irradiance of 80 μmol of photons m^-2^ s^-1^. Cultures were then transferred into 100 ml round bottles and the headspace was exchanged with Argon gas for anaerobiosis. Cells were harvested at an OD_660_ = 0.5, in mid-logarithmic phase, pelleted and stored at −20 °C. DNA was isolated from the cells using a CTAB bacterial genomic DNA isolation method, and checked on agarose gel. The genomic DNA purity was assessed by spectrophotometric measurements [40].

### Genome sequencing and assembly

The draft genome sequence was generated using the Illumina technology. A Nextera XT DNA library was constructed and sequenced using Illumina MiSeq platform which generated 23,625,870 reads. After trimming, a total of 7,574,912 paired end reads were obtained and assembled into 308 high quality contigs (larger than 5419 bp each) using Abyss 1.0.0 software present on the Galaxy Orione server [41]. A summary of the project information is shown in Table 2.

**Table 2:** Project information

MIGS ID	Property	Term
MIGS 31	Finishing quality	High-quality Draft
MIGS-28	Libraries used	Paired-end Nextera XT DNA
MIGS 29	Sequencing platforms	Illumina MiSeq
MIGS 31.2	Fold coverage	366 ×
MIGS 30	Assemblers	Abyss version 1.0.0 (Galaxy/CRS4 Orione server)
MIGS 32	Gene calling method	Prokka version 1.4.0 (Galaxy/CRS4 Orione server)
	Locus Tag	AB661
	Genbank ID	LCZM00000000
	GenBank Date of Release	5 June 2015
	GOLD ID	Gs0114708
	BIOPROJECT	PRJNA283573
MIGS 13	Source Material Identifier	CSMA73/42
	Project relevance	Metabolic versatility (hydrogen production), Biotechnology

### Genome annotation

Genes were identified using the prokaryotic genome annotation software Prokka 1.4.0 [42] (Galaxy Orione server [40]). For gene finding and translation, Prokka makes use of the program Prodigal [43]. Homology searching (BLAST, hmmscan) was then performed using the translated protein sequences as queries against a set of public databases (CDD, PFAM, TIGRFAM) as well as custom databases that come with Prokka. Additional gene prediction analysis and functional annotation were performed within the CBS Bioinformatics Tools platform developed by the Technical University of Denmark (Table 3).

**Table 3:** Genome statistics*

Attribute	Value	% of total
Genome size (bp)	5,128,858	100.00
DNA coding (bp)	4,388,835	85.00
DNA G + C (bp)	3,369,731	65.74
DNA scaffolds	1	100.00
Total genes	4767	100.00
Protein coding genes	4715	98.91
RNA genes	52	1.09
Pseudo genes	NA	NA
Genes in internal clusters	NA	NA
Genes with function prediction	3277	68.74
Genes assigned to COGs	3660	76.78
Genes with Pfam domains	3312	69.48
Genes with signal peptides	449	9.41
Genes with transmembrane helices	1212	25.42
CRISPR repeats	1	0.09

*NA, not available

## Genome properties

The genome of *R. palustris* 42OL resulted to be 5,128,858 bp in length with a GC content of about 65.74 % (Table 3). It was predicted to contain 4767 genes, 4715 of which coded for proteins and 52 for RNA (tRNA and rRNA). The majority of the predicted genes (68.74 %) could be assigned to one of of 25 functional COG categories whilst the 8.42 % of the remaining genes were annotated as hypothetical and 38.9 % as unknown function proteins. The distribution of genes into COGs functional categories is presented in Table 4.

**Table 4:** Number of genes associated with general COG functional categories

Code	Value	% age	Description
J	170	3.61	Translation, ribosomal structure and biogenesis
A	0	0.00	RNA processing and modification
K	218	4.62	Transcription
L	144	3.05	Replication, recombination and repair
B	1	0.02	Chromatin structure and dynamics
D	25	0.53	Cell cycle control, Cell division, chromosome partitioning
V	57	1.21	Defense mechanisms
T	192	4.07	Signal transduction mechanisms
M	215	4.56	Cell wall/membrane biogenesis
N	80	1.70	Cell motility
U	37	0.78	Intracellular trafficking and secretion
O	165	3.50	Posttranslational modification, protein turnover, chaperones
C	267	5.66	Energy production and conversion
G	169	3.58	Carbohydrate transport and metabolism
E	358	7.59	Amino acid transport and metabolism
F	59	1.25	Nucleotide transport and metabolism
H	145	3.08	Coenzyme transport and metabolism
I	239	5.07	Lipid transport and metabolism
P	242	5.13	Inorganic ion transport and metabolism
Q	97	2.06	Secondary metabolites biosynthesis, transport and catabolism
R	397	8.42	General function prediction only
S	383	8.12	Function unknown
–	1055	22.38	Not in COGs

The total is based on the total number of protein coding genes in the genome

## Insights from the genome sequence

The genome of *R. palustris* 42OL contained, as expected, genes related to nitrogen fixation (*nif* H, D, K, E, N, B, U, X, Q, W, Z), genes involved in carbon fixation (RubisCO), the complete tricarboxylic acid cycle, the glyoxylate shunt, a Embden-Meyerhof pathway, and a pentose phosphate pathway. Genes coding for the synthesis of glycogen and poly-β-hydroxyalkanoates as carbon storage polymers were also found, as well as genes related to the photosynthetic apparatus, similarly to all the other *R. palustris* strains so far sequenced.

The genome of *R. palustris* 42OL was analyzed in terms of synteny with other strains sequenced. The genome was found to be highly syntenic with those of other strains of *R. palustris* (data not shown). Exclusive reactions were then mapped on KEGG with respect to other *R. palustris* strains sequenced so far (BisA53, BisB18, BisB5, CGA009, Haa2, TIE1) by using DuctApe v 0.17.2 software [44]. Data obtained are reported in Table 5. The proteome size ranged between 4392 and 5242 protein coding genes, corresponding respectively to strains BisB5 and TIE1. The total number of reactions ranged between 2442 and 3012, respectively for strains BisA53 and CGA009. Strain Haa2 resulted to have the highest number of unique reactions.

**Table 5:** DuctApe analysis report

Strain ID	Proteome size	Mapped to KEGG	Reactions	Exclusive
420L	4715	2383	2775	0
BisA53	4851	2297	2442	5
BisB18	4864	2452	2523	19
BisB5	4392	2266	2787	4
CGA009	4811	2542	3012	0
Haa2	4680	2446	2881	31
TIE1	5242	2566	2984	0

### Conclusions

In this study, we characterized the genome of *R. palustris* strain 42OL isolated from a wastewater pond of a sugar refinery in 1973. Along the last four decades, this strain has been successfully used in a wide number of applications, from hydrogen production on wastewaters (its major application) to PHB production. The present genome analysis supported those findings.

## Abbreviations

CSMA: Centro Studi Microrganismi Autotrofi
CTAB: Cetyl trimethyl ammonium bromide
ICM: intra-cytoplasmic membrane
PHB: poly-β-hydroxy butyrate
PNSB: purple non sulfur bacterium
SCP: single cell protein
